# A Comparative Metabolomics Analysis Reveals the Tissue-Specific Phenolic Profiling in Two *Acanthopanax* Species

**DOI:** 10.3390/molecules23082078

**Published:** 2018-08-20

**Authors:** Ke-Xin Wu, Jia Liu, Yang Liu, Xiao-Rui Guo, Li-Qiang Mu, Xiao-Hang Hu, Zhong-Hua Tang

**Affiliations:** 1 Key Laboratory of Plant Ecology, Northeast Forestry University, Harbin 150040, China; wukexin94@126.com (K.-X.W.); liujia19880906@163.com (J.L.); yangyang1990520@163.com (Y.L.); xruiguo@nefu.edu.cn (X.-R.G.); 2 School of Forestry, Northeast Forestry University, Harbin 150040, China; 3 Crop Research Institute of Heilongjiang University, Harbin 150080, China

**Keywords:** *Acanthopanax*, phenolic compounds, UPLC-Q-TOF-MS, tissue-specificity

## Abstract

*Acanthopanax senticosus* (Rupr. Maxim.) Harms (ASH) and *Acanthopanax sessiliflorus* (Rupr. Maxim.) Seem (ASS), are members of the Araliaceae family, and both are used in Asian countries. These herbals have drawn much attention in recent years due to their strong biological activity, with innocuity and little side effects. However, the common and distinct mode of compound profiles between ASH and ASS is still unclear. In this study, a high performance liquid chromatograph-mass spectrometry (HPLC-MS) method was developed to simultaneously quantify the seven major active compounds, including protocatechuate, eleutheroside B, eleutheroside E, isofraxidin, hyperoside, kaempferol and oleanolic acid. Then the targeted metabolomics were conducted to identify 19 phenolic compounds, with tight relation to the above mentioned active compounds, including nine C6C3C6-type, six C6C3-type and four C6C1-type in the two *Acanthopanax* species studied here. The results showed that the seven active compounds presented a similar trend of changes in different tissues, with more abundant accumulation in roots and stems for both plants. From the view of plant species, the ASH plants possess higher abundance of compounds, especially in the tissues of roots and stems. For phenolics, the 19 phenols detected here could be clearly grouped into five main clusters based on their tissue-specific accumulation patterns. Roots are the tissue for the most abundance of their accumulations. C6C3C6-type compounds are the most widely existing type in both plants. In conclusion, the tissue- and species-specificity in accumulation of seven active compounds and phenolics were revealed in two *Acanthopanax* species.

## 1. Introduction

*Acanthopanax* is widely distributed in China, Korea and Japan [1]. In China, it is commonly used as ingredients of folk medicines, which have been officially listed in the Chinese Pharmacopoeia for a long time. In Western medicine, it is also widely used and included in the European Pharmacopoeia [2]. *Acanthopanax senticosus* Harms (ASH) and *Acanthopanax sessiliflorus* Seem (ASS) both belong to this genus, often called Ciwujia and Duangengwujia in China, respectively [3]. Various bioactive ingredients, i.e., triterperoid, saponins, lignans, flavones and phenolic compounds, exist in all parts of ASH and ASS plants [4,5,6]. Among them, elutheroside B is the main effect compound, which has immunomodulatary, antioxidant and anti-inflammatory activities [7,8]. Elutheroside E is another pharmaceutically active ingredient that shows stress protective activities [9]. Isofraxidin has antifatigue, antistress and immune-accomondating effects [10]. Caffeic acid, chlorogenic acid, rutin and kaempferol are the main phenolic compounds, which have obvious anti-oxidant activities [11]. Additionally, this herb is regarded to have a similar pharmacological function with that of Chinese ginseng [12]. Therefore, the quality control and evaluation of ASH and ASS plants recently become focus via comprehensive analysis of the multiple active ingredients. Plant phenolic compounds are the most widely distributed secondary metabolites, originating from the shikimate, phenylpropanoid and acetate metabolic pathways [13,14]. These compounds commonly have important functions in combating chronic diseases, such as 2-type diabetes, heart disease and various cancers, because of their high antioxidant activities [15,16,17]. In plants, they are also involved in the interactions between plant growth and environments [18]. Phenolic compounds contain at least one hydroxyl group attached to an aromatic ring and, according to the number and binding position of convertible hydroxyl groups on the aromatic chain, the plant phenolic compounds can be divided into three major groups: C6-C1-type (compounds have a C6-C1 carbon skeleton, such as hydroxybenzoic acids); C6-C3-type (compounds have a C6-C3 carbon skeleton, such as hydroxycinnamic acids); C6-C3-C6-type (compounds have a C6-C3-C6 carbon skeleton, such as flavonoids) [19,20,21]. For example, phenolic compounds are rich in olive leaves, including simple phenols and acids, lignans, secoiridoids and flavonoids [22].

In recent years, the high throughput approaches for profiling metabolic composition and levels have been increasingly applied in in plants [1,7,23,24]. Mass spectrometry methods have been used for detection and quantification of metabolites [25,26,27]. Ultra-performance liquid chromatography with quadrupole-time-of-flight mass spectrometry (UPLC-Q-TOF-MS) has been used in systems analysis of complicated metabolome [24,28,29]. A total of 37 compounds in *Lycopus lucidus* Turcz, including 15 phenolic acids, 12 flavonoids, three triterpenoids and seven organic acids were tentatively characterized and identified by means of the UHPLC-Q-TOF-MS method [30]. In the same way, 42 polymethoxylated flavonoids, including 33 flavones and nine flavanones, were distinguished in *Citrus reticulata* and *Citrus sinensis* [30,31]. This method was previously applied in *Acanthopanax senticosus* plants for the simultaneous determination of five major bioactive constituents (eleutheroside B, chlorogenic acid, caffeic acid, eleutheroside E and isofraxidin) [11]. The contents of chlorogenic acid and caffeic acid were investigated and validated in different parts of *Acanthopanx senticosus* and *Acanthopanx koreanum* by HPLC [3]. A total of 104 compounds have been identified in *Acanthopanx senticosus* fruit, including lignans, flavones, triterpenoid saponins, phenolic acids, and other constituents [32].

However, there is currently little information available about the tissue-specific metabolic profiling compared in *Acanthopanax senticosus* and *Acanthopanax sessiliflorus*, regarding their main effect compounds and phenolics. Therefore, we here targeted the major active compounds and phenolics, depicting their accumulations in these two plants. The metabolomics strategies and correlation analysis were then employed to dissect the species- and tissue-specific properties of 7 active compounds and 19 phenolics with medicinal values. Some potential marker compounds were screened out for identification of different species and determination of high accumulation of major effect compounds in *Acanthopanax* plants.

## 2. Results

### 2.1. Method Validation

**Figure 1:**
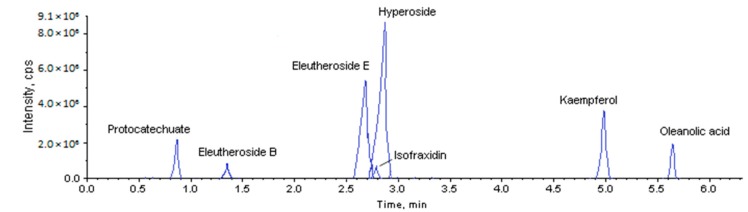
UPLC-MS chromatograms of 7 standard target compounds.

As shown in Figure 1, we obtained the representative total ion current (TIC) spectra for seven major constituents detected within 6 min. The retention times of protocatechuate, eleutheroside B, eleutheroside E, isofraxidin, hyperoside, kaempferol and oleanolic acid were found to be approximately 0.86, 1.37, 2.69, 2.76, 2.88, 5.05 and 5.66 min, respectively. The calibration curves, LOQs and LODs of all analytes are summarized in Table S1. All calibration curves correlation coefficient (r) were greater than 0.999. The LOD and LOQ values of the protocatechuate, hyperoside, isofraxidin and kaempferol were 0.045 ng/mL and 0.1 ng/mL, respectively, and the LOD and LOQ values of the oleanolic acid, eleutheroside B, and eleutheroside E were 0.45 ng/mL and 1 ng/mL, respectively. These results showed good linear relationships between peak area and concentration. To check the precision, accuracy and repeatability of this method, we injected the standard solutions of low, medium, and high concentration levels for inspection (Table S2). The results explain, with intra-day and inter-day precision (RSD) variations of 0.14% to 1.71% and 0.21% to 1.38%, and accuracy (RE) ranged from −1.03% to 0.95% and −1.02% to 1.69% respectively. Additionally, the repeatability (RSD) ranged between 0.29% and 1.92%. Thus, the developed method was considered to be reproducible and accurate. Based on these results, a simple and reliable UPLC-MS method for the quantification of the target compounds in the ASH and ASS has been developed and validated for its linearity, accuracy, precision, limits of detection, and limits of quantification.

### 2.2. Accumulation of Pharmaceutically Active Compounds in Two Acanthopanax Species

**Figure 2:**
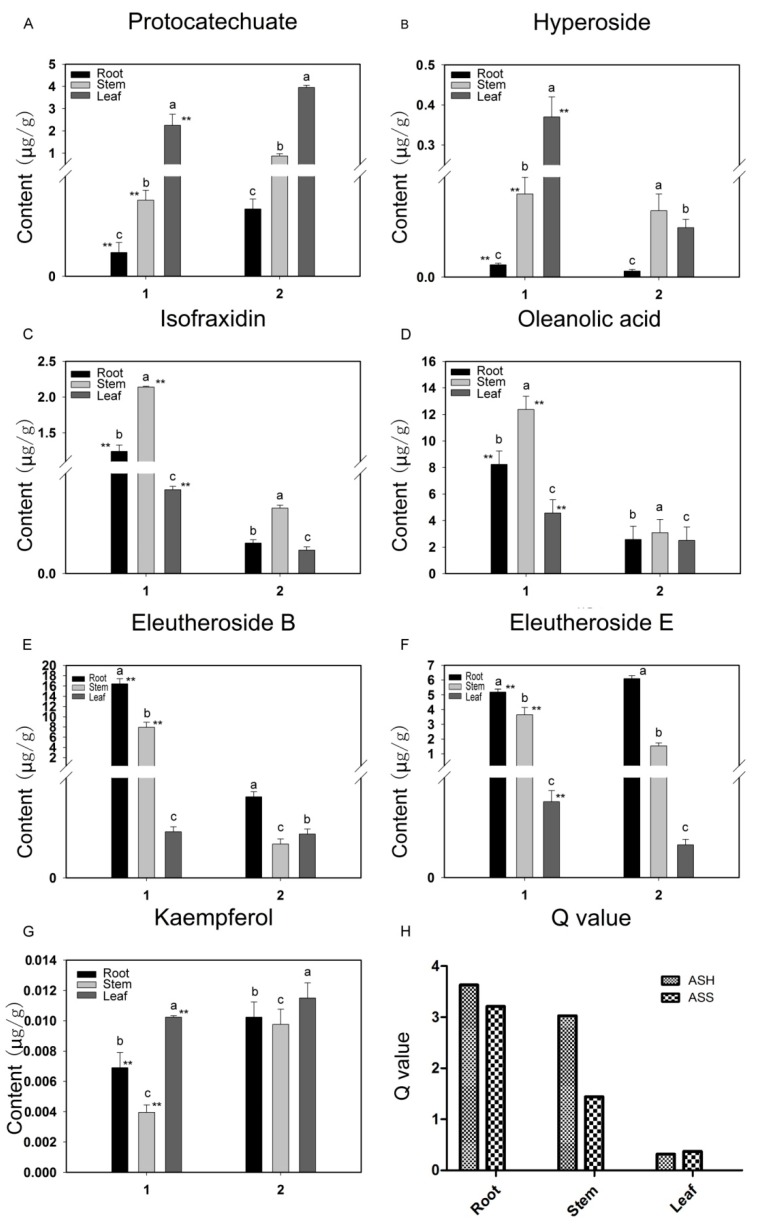
The contents of 7 major bioactive compounds in different parts of ASH and ASS (**A**–**G**) and the corresponding Q value (**H**). 1: ASH; 2: ASS. The letters a, b, c indicate significant differences between different tissues of the same plant (*p* < 0.05); ** indicates significant differences between different tissues of different plants (*p* < 0.01).

We then performed comparative analysis of seven active ingredients in ASH and ASS (Figure 2). The quantitative analysis was performed by means of the external standard methods. As shown in Figure 2, seven compounds displayed similar trends in different tissues of two species, although their contents were distinguished. The amount of hyperoside, isofraxidin, oleanolic acid and eleutheroside B in the roots of ASH were exhibited to be higher than ASS. In the stems, hyperoside, isofraxidin, oleanolic acid, eleutheroside B and eleutheroside E had significantly higher accumulation in ASH. However, protocatechuate and kaempferol contents were observed in the leaf of ASS were higher than in ASH. Eleutheroside B is an extremely important active substance in ASH [33]. Among the different tissues, the highest accumulation was observed in roots (16.406 μg/g) and leaves (7.940 μg/g) of ASH. Similarly, eleutheroside E is also the important active ingredient, exhibiting the highest levels at 6.091 μg/g and 5.191 μg/g, in roots of ASS and ASH plants, respectively. All original data for seven compound contents are showed in Table S3.

The principal component “Q” is an indicator of a comprehensive, scientific evaluation of objective phenomenon [34]. As shown in Figure 2H, the comprehensive Q value reflecting accumulation abundance of seven compounds in roots, stems and leaves of ASH and ASS were obtained. We can see from the Q value that the most abundant accumulation of the seven active compounds is in the roots and stems. From the view of plant species, the ASH plants possess higher abundance of compounds, especially in the tissues of roots and stems. From this result, it is clearly observed that the active ingredients show distinct species-specific and tissue-specific properties.

### 2.3. Targeted Analysis of Phenolic Compounds in Different Tissues of ASH and ASS

**Figure 3:**
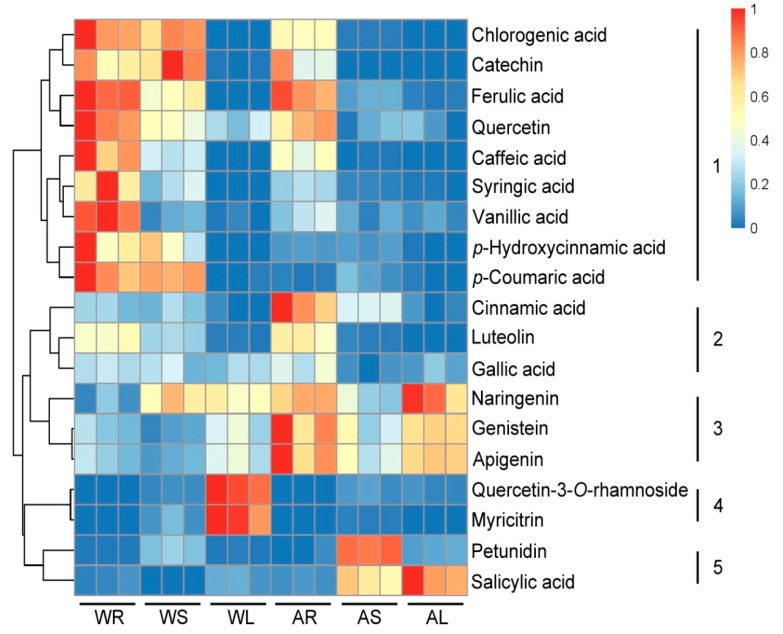
Heat map visualization of phenolics in different tissues of ASH and ASS. The color range from red to blue indicates relative abundance from high to low (color key scale right the heat map). Each sample was three repeated. W: ASH; A: ASS; R: root; S: stem; L: leaf.

Subsequently, we investigated the accumulation of phenolics in different tissues of ASH and ASS. Visualization of the phenolics was performed by hierarchical cluster analysis (HCA, Figure 3). Accumulation of phenolics displayed a clear variation in terms of their abundance in different tissues. Based on their tissue-specific accumulation patterns, the 19 phenols detected here could be clearly grouped into five main clusters. Phenolics in cluster 1 showed highest level mainly in WR and 3 types of compounds all accumulated in this cluster, including some major phenolics such as C6C1-type compounds (syringic acid, vanillic acid), C6C3-type compounds (chlorogenic acid, ferulic acid, *p*-coumaric acid), C6C3C6-type compounds (catechin, quercetin), respectively. Similarly, 3 types of compounds also occurred in cluster 2, including C6C1-type compound (gallic acid), C6C3-type compound (cinnamic acid) and C6C3C6-type compound (luteolin), at high level detected in AR. In cluster 3 and cluster 4, all compounds belong to C6C3C6type. The compounds of cluster 3 were substantially accumulated in ASS. Naringenin was mainly detected wth higher levels in AL, whereas high genistein and apigenin mainly in AR. However, myricitrin and quercetin-3-*O*-rhamnoside in cluster 4, showed a WL specific accumulation pattern. It is worth noting that, petunidin and salicylic acid were specifically present high accumulation in the AS and AL, respectively. As shown in Figure 3, the accumulation of phenolic compounds in ASH and ASS displayed significant species and tissue dependent specificity. Roots are the tissues containing the most abundance of phenolics, grouped to cluster 1 for ASH, whereas belonging to cluster 1, 3 and 4 for ASS. The data of 19 phenolic compound levels was in Table S4.

### 2.4. The Under-Ground and Above-Ground Distribution of Metabolites in the Specific Metabolic Pathways

**Figure 4:**
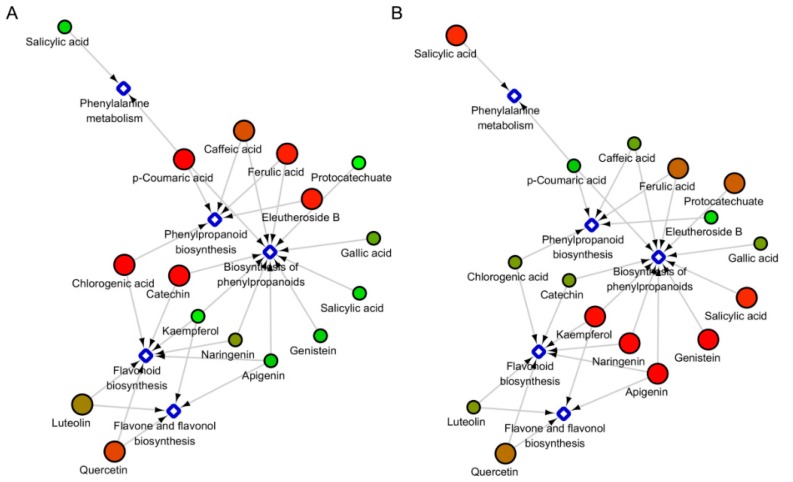
The associated network map of metabolites in ASH and ASS. The circles and diamonds nodes represent different metabolites and related metabolic pathways. Red indicates high abundance, whereas low relative phenolic compounds are blue. From red to green indicate abundance from high to low. Phenylpropanoid biosynthesis, Biosynthesis of phenylpropanoid, Flavonoid biosynthesis and Flavone and flavonol bilsynthesis are mutually different metabolic pathways. (**A**) ASH; (**B**) ASS.

To gain a general view of these metabolites in terms of tissue- and species-specificity, we visualized the network map of metabolic pathways based on their accumulation ratio in the under- and above-ground tissues (Figure 4). The results revealed that 15 compounds were involved in four metabolic pathways, including phenylalanine, flavonoid, flavonol and phenylpropanoid, respectively. It is worth noting that the phenylpropanoid biosynthesis pathway consists of two parts. We observed a significant discrepancy in the two species, with the major part of compounds displaying contrasting trend. The major active ingredient, eleutheroside B, participates in the phenylpropanoid biosynthesis pathway, and it mainly accumulated in the underground part of ASH. Some phenolic compounds such as *p*-coumaric acid, caffeic acid, ferulic acid, chlorogenic acid and catechin also had the same accumulation pattern. However, in ASS undergraduate tissues, abundantly accumulated compounds mainly involved the active ingredient kaempferol, as well as the phenolic compounds naringenin, apigenin, genistein and salicylic acid, and they separately participate in pathways of flavonoid, flavone, flavonol and phenylpropanoid biosynthesis.

### 2.5. Correlation Analysis of 7 Active Compounds and 19 Phenols in ASH and ASS

**Figure 5:**
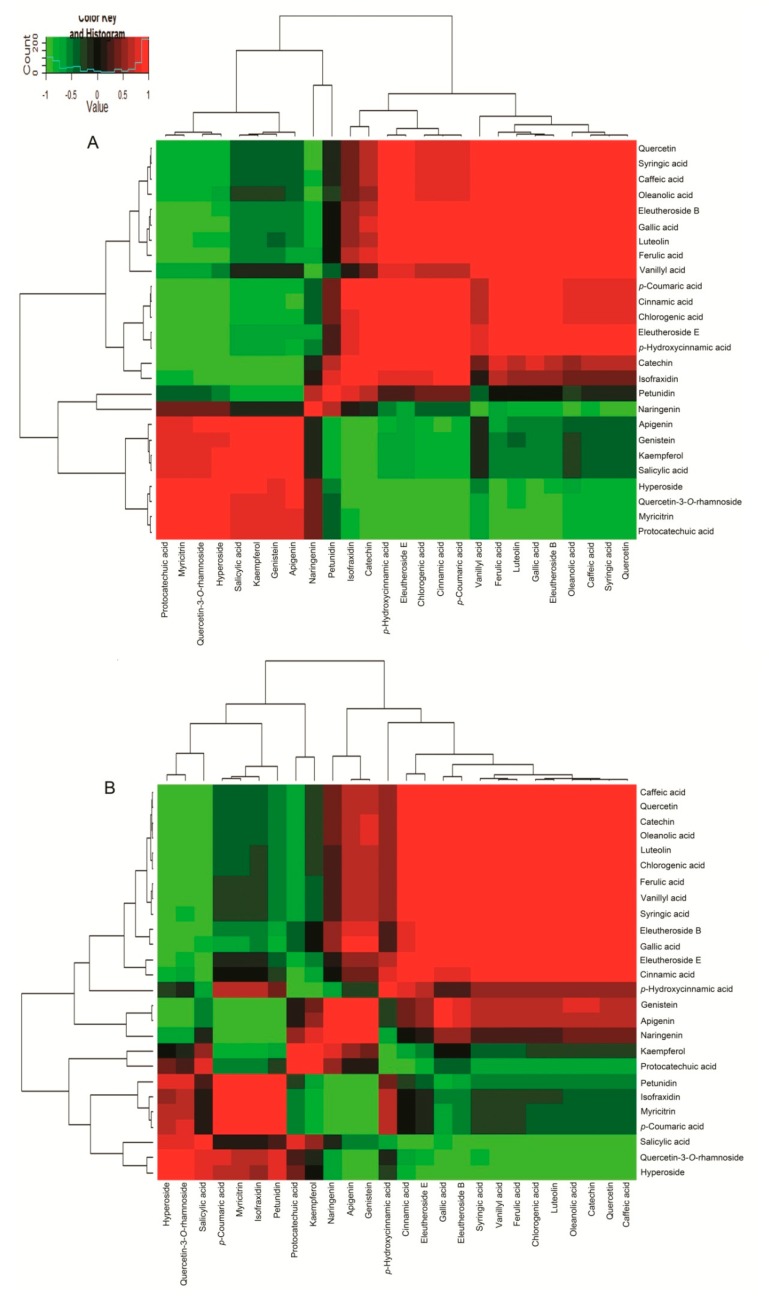
Correlation coefficients of the metabolites analyzed with 7 bioactive compounds and 19 phenolic compounds in different plants. Each square indicates r (Pearson’s correlation coefficient value for pairs of metabolite). The red color represents a negative correlation and the green color represents a positive correlation. (**A**) ASH; (**B**) ASS.

To further study the relationship among metabolites, the total 26 compounds were subjected to correlation assay. As shown in Figure 5, eleutheroside B showed positive correlation with luteolin (*p* < 0.05) and ferulic acid (*p* < 0.05) in ASH, however, it displayed positive correlation with C6C1-type compound gallic acid (*p* < 0.05) in ASS. In ASH, protocatechuate showed negative correlation with chlorogenic acid (*p* < 0.05) and *p*-coumaric acid (*p* < 0.05) of C6C3-type compounds. Kaempferol displayed negative correlation with apigenin (*p* < 0.05), genistein (*p* < 0.05) and salicylic acid (*p* < 0.01). Caffeic acid positively correlated with catechin (*p* < 0.01), luteolin (*p* < 0.05) and chlorogenic acid (*p* < 0.05) in ASS. Naringenin displayed negative correlation with *p*-coumaric acid (*p* < 0.05). These results were consistent with the information available in network map of metabolites (Figure 4).

Additionally, the C6C3C6-type compound quercetin-3-*O*-rhamnoside displayed negative correlation with cinnamic acid (*p* < 0.05), *p*-coumaric acid (*p* < 0.05) and chlorogenic acid (*p* < 0.05) of C6C3-type compounds in ASH. In ASS, hyperoside showed a negative correlation with eleutheroside B and the C6C1-type compound gallic acid (*p* < 0.01). Salicylic acid (C6C1) displayed negative correlation with eleutheroside E (*p* < 0.05), while positive correlation with ferulic acid (*p* < 0.05) of C6C3-type compounds. It was interesting to find that *p*-coumaric acid and myricitrin (*p* < 0.01) showed negative correlation in ASH, however, they displayed positive correlation (*p* < 0.05) in ASS.

## 3. Discussion

The accumulating mode of active compound biosynthesis in *Acanthopanax* plants regulated by heredity and environments is increasingly a focus problem. As one of traditional medicinal plants in northeast of China, ASH and ASS plants produce large number of active compounds [1,35]. Increasing evidence proposes that overall pharmacological activities of ASH lie more on the combination of multiple substrates, other than only one or two constituents [3,10]. Therefore, it is necessary to establish a more comprehensive quality inspection method to detect a variety of constituents [36,37]. In order to make more rational use of medicinal active ingredients and protect *Acanthopanax* resources, this study explores the tissue-specific distribution of medicinal ingredients in ASH and ASS and provides instruction on how efficiently utilize *Acanthopanax* plants.

In this study, we first developed an efficient UPLC-MS method to simultaneously detect 7 major target compounds in ASH and ASS. Compared with the time-consumed HPLC method, the UPLC-MS provide faster separation, higher resolution and higher sensitivity for analysis and elucidation of drug metabolism [13,38,39,40]. Preciously, a method for the simultaneous determination of major bioactive components in *Acanthopanax senticosus* (protocatechuic acid, eleutheroside B, chlorogenic acid, caffeic acid, lithium complex and isocarotenoid) by HPLC was established in 40 min [1]. Here, the preparation method of plant samples is optimized, and the extraction method by methanol extraction is effective. The MS ion mode, MS parameters, and mobile phase for all analytes were optimized for higher MS sensitivity and better peak shape. After repeated tests, we found that in the positive ion mode 0.04% acetic acid and the mobile phase methanol-water was better than other solutions. In this condition, we greatly improved the speed and quality of the detection (Figure 1; Supporting Information Tables S1 and S2).

As shown in Figure 2, among the seven active compounds, the content of eleutheroside B is higher in ASH than other metabolites. In contrast, eleutheroside E had a high accumulation in the roots of ASS. Q value analysis of the seven active compounds tested here found their accumulation is significantly higher in ASH than in ASS, especially in the roots and stems. This indicates that ASH may be superior to ASH for some pharmacological effects, and needs further exploration. Previous studies have mostly focused on the roots and stems while the leaves are often used as dietary supplements [10,41]. Our results showed the leaf tissue also contain some important metabolites with high levels, indicating that the leaf may have medicinal value to be further developed.

Phenolics are important secondary metabolites in plant, always displaying a tissue-specific distribution in the content and composition [14,42,43]. Targeted analyses were performed to identify 19 phenolic compounds in our study (Figure 3). The C6C3C6-type was the most accumulated compound. It indicates that the C6C3C6 compounds are mainly involved in plant biosynthesis of flavonoid and plays a role of anti-ultraviolet radiation [44]. It is noteworthy that the three C6C3C6-type compounds have obvious tissue-specific property, including quercetin-3-O-rhamnoside, myricitrin and petunidin. This result shows that we can target these marker active ingredients in different tissues during cultivation and extraction of them. The compound *p*-coumaric acid is known to be an effective antioxidant responsible for ABTS free radical scavenging activity [45]. It shows that ASH has better ABTS radical scavenging ability than ASS, and has better oxidation resistance. The previous study found that ferulic acid and quercetin-3-*O*-rhamnoside is considered to have well anticancer properties [46]. Therefore, we speculate that in the anti-cancer ability, ASH may be better than ASS. Salicylic acid has the effect of reducing the risk of long-term heart disease in diabetic patients and is only present in ASS [47,48]. When treating such diseases, ASS can be selected as the experimental material.

During the construction of related network maps of metabolites in ASH and ASS, the accumulation patterns of 15 metabolites involved in the four metabolic pathways in different plants were obtained (Figure 4). The biosynthetic pathway of the C6C3C6 compound results in the production of flavonoids (flavonols) and isoflavones [49,50]. However, C6C1 compounds may play important roles mainly as signaling molecules such as benzoic acid derivatives and salicylic acid [51]. All compounds have different or completely opposite patterns of accumulation in ASH and ASS. According to this comparison, we can provide an alternative way to identify ASH and ASS, by the ratio of target compounds between the aboveground and underground parts of plants.

Combined with the metabolic network map and metabolites correlation analysis, it can be concluded that the correlation between compounds may affect accumulation of them in different species [52]. It is worth noting that eleutheroside B with luteolin and ferulic acid mainly accumulated in the underground part in ASH, and they had significant correlation; in ASS, eleutheroside B had a significant correlation with gallic acid and they had plenty of accumulations in the aboveground part (Figure 5). The C6-C3 branching pathway metabolites, namely *p*-coumaric acid, ferulic acid and trans-cinnamic acid, are reported to be grouped with salicylic acid [20]. We proposed that eleutheroside B might be mainly regulated by luteolin and ferulic acid in ASH and it was controlled by gallic acid in ASS. The phenolic compounds as reactive oxygen scavengers, disease-inducing agents had important functions in plant development and defense [53,54]. For the correlation analysis of all compounds, we found that in ASS the active compounds eleutheroside B, hyperoside and eleutheroside E and were correlated with the C6-C1 compounds, while in ASH, the active compounds eleutheroside B, eleutheroside E, oleanolic acid, protocatechuate and hyperoside were associated with C6-C3 compounds. These results indicate that the crucial components determining the same active compounds might be different in different plants. 

In summary, we successfully developed a sensitive and efficient UPLC-MS method to simultaneously determine 7 active metabolites in ASH and ASS. The 19 phenolic compounds from these two plants were also targeted and analyzed. In the two plants, eleutheroside B, eleutheroside E and oleanolic acid are the most abundant compounds. The Q value of total compound profiles was significantly higher in ASH than in ASS whether in roots, stems. Roots are the most abundant tissue for accumulation of phenolic compounds, either in ASH or ASS. C6C3C6-type compounds are the most widely existing type in both plants. It was considered that the C6C1-type compounds were more capable of modulating the active ingredient in the ASS, while in ASH it was C6C3-type compound. In comparison of two plants, the ratio of phenolic compounds between the aboveground and underground parts had great discrepancy. We propose ASH and ASS plants can be more efficiently used for different objective in light of containing abundance of tissue- and species-specific active compounds.

## 4. Materials and Methods

### 4.1. Chemicals and Reagents

UPLC-grade acetonitrile and methanol were purchased from Fisher Scientific (Pittsburgh, PA, USA). All other reagents were of analytical purity. Ultrapure water was prepared by w Milli-Q system (Millipore, Bedford, MA, USA) water purification system. The reference compounds required for the experiment were all purchased from ChromaDex Inc. (Santa Ana, CA, USA), including eleutheroside B, eleutheroside E, protocatechuate, hyperoside, oleanolic acid, kaempferol, isofraxidin, syringci acid, vanillic acid, salicylic acid, cinnamic acid, gallic acid, ferulic acid, cholorogenic acid, caffeic acid, *p*-hydroxycinnamic acid, *p*-coumaric acid, petunidin, luteolin, apigenin, naringenin, genistein, quercetin-3-*O*-rhamnoside, quercetin, myricitrin and catechin. The purities of these standards were higher than 98%.

### 4.2. Sample Preparation

The cultivated ASH and ASS plants (2-year-old) were collected from our field base in Bajiazi Forestry Bureau of Jilin province located in Northeast of China (129.12′ E, 42.67′ N) in 2016. Three independent biological samples were collected on 10 plants per species randomly selected in our experimental plot. The whole plant was dissected to obtain roots, stems and leaves for metabolic analysis. The raw materials were dried at 60 °C for 48 h. The experiment performed three replications.

Dried leaf, stem, and root of ASH and ASS were ground in a mill and passed through a 35-mesh sieve. Approximately 2 g of dry powdered plant material was extracted with 10 mL of methanol (80%) by reflux for 45 min. The extract was repeatedly filtered and the filtrate was collected. The extract was subjected to centrifugation at 14,000 rpm at 4 °C for 10 min. The supernatant was moved out, and the extract was concentrated by evaporation under a vacuum to dryness. Then the precipitate was dissolved with methanol to a volume of 1.0 mL. All samples were filtered through micropores filter membrane of 0.22 μm diameters, which could be directly injected for LC-MS analysis.

### 4.3. Instrumentation and Conditions

Measurement of the major effect compounds were carried with the UPLC-MS system, containing an ultra-performance with a LC-20AD pump, a temperature controller and column oven. The chromatographic separation was performed on an Acquity UPLC BEH C18 (1.7 μm, 2.1 mm × 5 mm) column. The column temperature was maintained at 25 °C and the injection volume was 5 μL. The elution gradient of the mobile phase consist of water and methanol at a flow rate of 0.25 mL/min: 25% methanol at 0.0–1.5 min, 25–50% methanol at 1.5–2.0 min, 50% methanol at 2.0–4.0 min, 50–90% methanol at 4.0–4.5 min, 90% methanol at 4.5–5.5 min, 90–25% methanol at 5.5–6.0 min, 25% methanol at 6.0–7.0 min. Mass spectrometry was carried out on a QTRAP 5500 Ion TRAP MASS Spectrometer (AB SCIEX, Boston, MA, USA) equipped with an electrospray ionization source that was operated in positive ion mode. The experimental conditions were as follows: ion spray voltage, 3000 V; turbo spray temperature, 500 °C; high purity nitrogen was used in all units; pressure of nebulizer gas, 25 psi; curtain gas, 15 psi. Seven metabolite fragment ions, cluster voltage, collision voltage and chamber injection voltage are provided in Table 1. This method is used for the detection of 7 active ingredients and data was utilized with Analyst 1.4.2 software version (AB SCIEX, Concord, ON, Canada).

Then, the UPLC-QTOF-Tandem mass spectrometer via electrospray ionization (ESI) interface (Waters G2, Milford, MA, USA) were utilized to analyze the targeted 19 phenolic compounds. Analysis was conducted on the gradient elution component using solvent A (0.04% formic acid-water) and solvent B (0.04% Formic acid-acetonitrile) as the mobile phase. The gradient elution with the flow rate of 0.5 mL/min was performed as follows: 0.0–20.0 min, 5–95% B; 20.0–22.1 min, 95–5% B; 22.1–28.0 min, 5% B. The full-scan data was acquired in the positive ion mode, the MS conditions were set as follows: scan time 1.0 s, mass range from 50 to 1000 *m*/*z*, fragmentation voltage 105 V, capillary voltage 3500 V, source temperature was set a 350 °C, curtain gas pressure of 40 psi. In an environment of 25 °C, chromatographic separations were achieved on an Acquity UPLC BEH C18 column (1.7 μm, 2.1 mm × 5 mm), the volume injected was 5 μL. The phenolic data were acquired with MassLynx software v 4.1 (Waters, Milford, MA, USA).

### 4.4. Data Processing and Statistical Analysis

Statistical analysis of data was performed using SPSS 17.0 software (SPSS, Inc., Chicago, IL USA). The results were expressed as mean values (mean ± standard error), and the difference between the samples were compared by using a multiple-range test at *p* < 0.05 probability level (Duncan’s test). The total 19 phenolic compounds were subjected to the heat map and hierarchical clustering analysis by R Language (www.r-project.org/).

## Figures and Tables

**Table 1:** Transitions, cluster voltage, collision voltage and chamber injection voltage of seven compounds.

Compounds	*m*/*z*	DP	CE	CXP
Oleanolic acid	479.3→435.5	70	28	17
Kaempferol	287.1→153	70	51	10
Isofraxidin	223.1→206.3	60	40	9
Eleutheroside B	394.8→231.8	70	40	17
Eleutheroside E	765.3→603.1	70	62	23
Hyperoside	487→324	70	43	17
Protocatechuate	155→92.9	70	21	17

## Supplementary Materials

The following are available online at, Table S1: The regression equations, linear range, LODs and LOQs the tested compounds, Table S2: The intra-day and inter-day precision, accuracy and repeatability analysis of Protocatechuate, Hyperoside, Isofraxidin, Oleanolic acid, Eleutheroside B, Eleutheroside E and Kaempferol at low, medium, and high concentration levels (*n* = 6), Table S3: The content of seven major bioactive compounds in different parts of ASH and ASS, Table S4: The data of 19 phenolic compounds heat map.
