# Supplementary material for: A Comparative Metabolomics Analysis Reveals the Tissue-Specific Phenolic Profiling in Two Acanthopanax Species
Source: Molecules. 2018 Aug 20;23(8):2078. doi: 10.3390/molecules23082078 (PMC6222473; doi:10.3390/molecules23082078)
Supplement: Supplementary file 1 [file molecules-23-02078-s001.pdf]

Table S1. The regression equations, linear range, LODs and LOQs the tested compounds

Metabolites	Range (ng/mL)	Calibration curves	Correlation coefficient (*r*)	Limit of detection (ng/mL)	Limit of quantitation (ng/mL)
Protocatechuate	0.1-1000	Y= 292.4x + 10111	0.9997	0.045	0.1
Hyperoside	0.1-1000	Y=6290.3x + 2E+06	0.9990	0.045	0.1
Isofraxidin	0.1-1000	Y=250.87x - 44.444	1.0000	0.045	0.1
Oleanolic acid	1-10000	Y=27.767x + 56290	0.9998	0.450	1.0
Eleutheroside B	1-10000	Y= 3241.1x - 9252.4	1.0000	0.450	1.0
Eleutheroside E	1-10000	Y = 502.3x + 30434	0.9997	0.450	1.0
Kaempferol	0.1-1000	Y=3031.7x - 8055.6	0.9999	0.045	0.1

Y indicates the peak area;X indicates the concentration.

Table S2. The intra-day and inter-day precision, accuracy and repeatability analysis of Protocatechuate, Hyperoside, Isofraxidin, Oleanolic acid, Eleutheroside B, Eleutheroside E and Kaempferol at low, medium, and high concentration levels (n=6).

Metabolites	Nominal concentrations (ng/mL)	Intra-day		Inter-day		Repeatability (relative standard deviation %)
		Precision (relative standard deviation %)	Accuracy (relative error %)	Precision (relative standard deviation %)	Accuracy (relative error %)	
Protocatechuate	8	0.82%	-0.45%	0.95%	1.69%	1.46%
	80	1.20%	-1.02%	1.20%	-1.02%	1.15%
	800	0.42%	-0.23%	0.46%	-0.36%	0.58%
Hyperoside	8	0.24%	0.15%	0.47%	-0.08%	0.61%
	80	0.37%	0.24%	0.65%	0.86%	0.32%
	800	1.33%	-0.16%	1.32%	0.73%	0.82%
Isofraxidin	8	0.51%	-1.03%	0.91%	0.37%	1.12%
	80	1.71%	0.95%	1.35%	0.56%	0.78%
	800	0.59%	-0.24%	1.38%	-0.56%	0.98%
Oleanolic acid	80	0.81%	0.33%	1.11%	0.42%	1.42%
	800	0.33%	0.14%	0.21%	-0.08%	0.75%
	8000	1.07%	-0.44%	1.30%	-0.53%	1.92%
Eleutheroside B	80	0.67%	-0.27%	0.50%	0.20%	1.00%
	800	0.14%	0.06%	0.86%	-0.35%	0.77%
	8000	0.60%	0.25%	0.90%	0.37%	0.45%
Eleutheroside E	80	0.74%	-0.40%	0.75%	0.31%	0.37%
	800	0.58%	0.30%	0.98%	0.40%	0.79%
	8000	0.96%	-0.48%	0.58%	0.24%	0.51%
Kaempferol	8	0.61%	0.25%	0.86%	0.35%	0.86%
	80	1.08%	-0.44%	0.54%	-0.22%	0.29%
	800	0.23%	0.00%	0.65%	-0.26%	1.13%

Table S3 The content of seven major bioactive compounds in different parts of ASH and ASS

		ASH			ASS		
	（μg/g）	Root	Stem	Leaf	Root	Stem	Leaf
1	Eleutheroside E	5.1908	3.6508	0.0683	6.0908	1.5408	0.0294
2	Oleanolic acid	8.2408	4.5741	3.0875	2.3741	2.5741	2.5075
3	Kaempferol	0.0069	0.0039	0.0102	0.0102	0.0098	0.0115
4	Isofraxidin	1.2402	2.1402	0.0251	0.0091	0.0196	0.0070
5	Protocatechuate	0.1189	0.3802	2.2516	0.3362	1.8716	3.9516
6	Eleutheroside B	16.4062	7.9395	0.0922	0.1622	0.0677	0.0875
7	Hyperoside	0.0007	0.0050	0.3700	0.0004	0.0040	0.0030

Table S4 The data of 19 phenolic compounds heat map

id	WR	WR	WR	WS	WS	WS	WL	WL	WL	AR	AR	AR	AS	AS	AS	AL	AL	AL
p-Hydroxycinnamic acid	1	0.4892	0.593851	0.718083	0.473857	0.282686	0	0.000186	0.000593	0.082479	0.098312	0.080404	0.098003	0.078175	0.095753	0.012603	0.005838	0.003095
Genistein	0.275723	0.193823	0.152284	0.049467	0.090409	0.119275	0.343194	0.420196	0.229634	1	0.624536	0.844395	0.532614	0.21469	0.321994	0.652038	0.695852	0.677862
Apigenin	0.297702	0.210683	0.157941	0.089644	0.1276	0.155193	0.359416	0.429223	0.254936	1	0.66025	0.825846	0.505048	0.273414	0.37079	0.672725	0.718611	0.693515
Syringic acid	0.614481	1	0.583805	0.154852	0.261	0.35115	0.002382	0.000859	0	0.219295	0.273204	0.249622	0.048206	0.044162	0.03936	0.014386	0.011507	0.005683
Petunidin	0.016037	0.01406	0.014375	0.176779	0.224053	0.182621	0.013654	0.02289	0.018278	0.003504	0	0.054358	0.885108	0.868362	0.908461	0.09807	0.115813	0.126859
Naringenin	0.048571	0.203501	0.065786	0.516026	0.743233	0.586333	0.596476	0.470991	0.499855	0.680056	0.774532	0.779227	0.428795	0.218165	0.193532	0.982959	0.89567	0.632991
Quercetin-3-*O*-rhamnoside	0.001795	0.007933	0	0.049375	0.065994	0.053567	1	0.946599	0.886501	0.009561	0.003876	0.008913	0.085117	0.10243	0.056629	0.071619	0.044187	0.040658
Chlorogenic acid	1	0.803851	0.781116	0.659277	0.849058	0.817315	0.008441	0.007413	0.007182	0.537655	0.494117	0.510119	0.02755	0.028241	0.026529	0	0.000433	0.000628
Salicylic acid	0.033783	0.046575	0.077342	0	0.000573	0.005254	0.129069	0.130317	0.073295	0.063376	0.085581	0.069496	0.716635	0.606847	0.53765	1	0.798515	0.765153
Ferulic acid	1	0.898987	0.910039	0.456444	0.49998	0.580536	0.001813	0	0.006787	0.949041	0.810463	0.756041	0.097818	0.130091	0.13455	0.039686	0.015201	0.023666
Myricitrin	0.001775	0.008033	0.009517	0.07197	0.163201	0.061593	1	0.971385	0.804506	0.004552	0.004706	0.004826	0.031275	0.02257	0.022378	0.003193	0.00126	0
Luteolin	0.478	0.460	0.527	0.233	0.253	0.217	0.024	0.023	0.014	0.585	0.594	0.478	0.041	0.027	0.024	0.000	0.003	0

	992	349	288	455	562	404	669	374	447	976	537	485	678	936	838	253	774	
Catechin	0.826<br>129	0.515<br>64	0.588<br>077	0.636<br>618	1	0.842<br>729	0.010<br>092	0.005<br>313	0.017<br>155	0.833<br>191	0.364<br>406	0.388<br>431	0.007<br>19	0.005<br>078	0.009<br>9	0.001<br>579	0	0.002<br>859
Cinnamic acid	0.236<br>783	0.241<br>952	0.155<br>344	0.144<br>229	0.267<br>003	0.182<br>172	0.050<br>491	0.008<br>624	0.002<br>041	1	0.824<br>412	0.691<br>37	0.337<br>879	0.349<br>625	0.356<br>092	0.082<br>099	0.007<br>904	0.047<br>669
p-Coumaric acid	1	0.830<br>964	0.718<br>588	0.786<br>693	0.753<br>815	0.793<br>545	0.002<br>221	0	0.024<br>138	0.034<br>392	0.013<br>523	0.028<br>28	0.174<br>336	0.109<br>818	0.068<br>765	0.027<br>27	0.009<br>485	0.004<br>835
Quercetin	1	0.852<br>725	0.802<br>991	0.508<br>87	0.480<br>412	0.405<br>166	0.251<br>836	0.168<br>391	0.322<br>097	0.564<br>5	0.751<br>38	0.800<br>472	0.013<br>291	0.127<br>82	0.188<br>435	0.198<br>349	0.089<br>93	0
Gallic acid	0.263<br>775	0.302<br>809	0.259<br>855	0.271<br>939	0.333<br>616	0.145<br>612	0.155<br>474	0.266<br>608	0.254<br>762	0.363<br>438	0.263<br>463	0.449<br>07	0.066<br>072	0	0.076<br>532	0.086<br>381	0.207<br>814	0.108<br>289
Caffeic acid	1	0.699<br>002	0.812<br>356	0.321<br>191	0.272<br>864	0.311<br>362	0.002<br>187	0.002<br>064	0.002<br>55	0.485<br>48	0.385<br>117	0.500<br>781	0.006<br>706	0.013<br>082	0.016<br>619	0.001<br>104	0	0.001<br>125
Vanillic acid	0.921<br>417	1	0.863<br>879	0.043<br>52	0.127<br>98	0.154<br>78	0.016<br>602	0.046<br>921	0	0.188<br>177	0.285<br>733	0.350<br>033	0.125<br>259	0.039<br>179	0.120<br>79	0.063<br>781	0.111<br>537	0.043<br>299
